# Transgenic Plant Detection Using an AuNPs Based SPR Biosensor

**DOI:** 10.3390/bios9040116

**Published:** 2019-09-30

**Authors:** Bartosz F. Grześkowiak, Karol Tuśnio, Anna Woźniak, Marlena Szalata, Daniel Lipiński, Stefan Jurga, Ryszard Słomski

**Affiliations:** 1 The NanoBioMedical Centre, Adam Mickiewicz University, Wszechnicy Piastowskiej 3, 61-614 Poznan, Poland; k.tusnio@gmail.com (K.T.); wozniaka@amu.edu.pl (A.W.); stjurga@amu.edu.pl (S.J.); 2 Department of Biochemistry and Biotechnology, Poznań University of Life Sciences, Dojazd 11, 60-632 Poznan, Poland; szalata@up.poznan.pl (M.S.); lipinskidaniel71@gmail.com (D.L.); slomski@up.poznan.pl (R.S.)

**Keywords:** biosensor, genetically modified organism (GMO), gold nanoparticles, surface plasmon resonance (SPR), transgene detection

## Abstract

The intensive development and commercialization of genetically modified plants observed over the last decade has led to the development of transgenic detection methods that are rapid and sensitive. Among the strategies used for the detection/monitoring of genetically modified organisms (GMOs), surface plasmon resonance (SPR) meets the necessary criteria. This optical technique measures the changes in the refractive index in the vicinity of thin metal layers (i.e., gold) in response to biomolecular interactions occurring at a flat metal‒solution interface. Additionally, it allows the application of functionalized gold nanoparticles (AuNPs) in SPR research to enhance the signal intensity. In the present study, an SPR method, enhanced by the application of AuNPs, was developed to detect transgenic tobacco plants carrying a *Streptococcus mutans* antigen. The basis for the detection of the target DNA was the hybridization between the genomic DNA isolated from the leaves, stems, and roots of the transgenic tobacco and the biotinylated oligonucleotide probes immobilized onto a streptavidin (SA) sensor chip. SA-functionalized AuNPs coated with a second type of biotinylated probe were applied to increase the sensitivity of the detection method. Analysis of the results indicated that the constructed SPR-based sensor chip can potentially recognize complementary standard fragments (nonamplified genomic DNA) at concentrations as low as 1 pM. Thus, nonamplified transgenic DNA was detected using a label-free and real-time AuNPs-enhanced SPR biosensing method. This unique approach could be used to detect GMOs with high efficiency, even at a low detection limit, high repeatability, and with less time and a lower cost needed for each analysis.

## 1. Introduction

Genetic modification refers to the introduction of an exogenous gene that can express a protein with a specific function. Genetically modified organisms (GMOs) exhibit new traits that can be used for a plethora of applications (e.g., biomedicine, agro-food science, and industry). The development and commercialization of GMOs and their related products will lead to their increased exposure to humans and the environment, and thus, the identification of GMOs may require greater attention.

There are two common types of strategies used for GMO detection. The first strategy is based on transgene product targeting indirect protein-based techniques and includes Western blotting, enzyme-linked immunosorbent assays, lateral flow strip tests, and mass spectrometry [1,2]. These techniques offer several advantages, such as their rapidity and simplicity. However, the protein-based methods depend on the expression level of the targeted proteins and a nondegraded structure and are not applicable if the genetic modification has no impact at the protein level [3]. To overcome these issues, a second approach, DNA-based methods, including polymerase chain reaction (PCR) [4], Southern blot [5], DNA microarrays [6], and next generation sequencing [7] technologies, has been developed. Among these techniques, quantitative PCR (qPCR) is the method of choice in the routine analysis of GMOs. A qPCR system allows the detection, identification, and quantification of GMOs with high accuracy and reliability [8]. However, the cost of the assay, the complexity of the detection, and the quantity of the DNA required for the assay are high due to the PCR amplification step. Therefore, there remains a need for the development of PCR-free detection methods that can perform fast DNA analyses with ultrasensitivity and high repeatability. It seems that methods based on surface plasmon resonance (SPR) can meet these requirements.

SPR is a physical phenomenon that occurs at the interface of two media (for example, a metal and a dielectric material). If the SPR sensor is illuminated at a right angle, a part of the photon energy of the incident beam can be transferred to excite the electrons oscillating in the metal. To allow this, the frequency of the photons of the incident light should be consistent with the frequency of the plasmon oscillations. Thus, it is possible to observe the loss of energy of the reflected light beam returning to the detector. Because the resonance conditions mainly depend on the refractive index units (metal/dielectric), even minimal changes in the vicinity of the metal surface can be observed. Any change in the refractive index of the medium leads to a change in the angle at which the conditions for the occurrence of the SPR phenomenon are fulfilled. Therefore, various types of interactions can be monitored in real time.

The Biacore X100 system allows label-free detection for monitoring the interactions between molecules. The detection is performed on a specifically designed chip covered with a thin layer of gold. Firstly, a corresponding ligand is immobilized on the functionalized surface of a sensor. The target analyte is then introduced into the solution to flow over the sensor surface. The binding of the molecules from the injected sample with the immobilized molecules results in a shift in refractive index at the surface of the sensor. This allows monitoring of the various types of interactions in real time. The results are presented in a sensorgram, which shows the binding curves exhibiting the association and dissociation rates of the interactions. The SPR signal intensity is measured in resonance units (RU). A change by 1 RU corresponds to a change in the SPR angle by 0.0001°, which indicates that 1 pg of particles are adsorbed on 1 mm^2^ of the sensor surface. This SPR-based biosensing approach can be widely applied in many fields, such as biology, clinical diagnostics, environmental safety, and agro-food testing [9,10,11,12]. An additional advantage is that the result can be obtained much faster compared with conventional diagnostic methods, with limited demand for reagents. A typical analysis cycle takes about 5‒15 min, and the sensor chips can be reused for several runs.

Although the SPR technology is a versatile biosensing method, its application in certain cases to detect specific types of interactions is limited. In the case of DNA or RNA samples, the hybridization process on the sensor’s surface may not be registered. To overcome this disadvantage, many works have been done to develop new procedures that allow enhancing of the SPR response during the detection of nucleic acids [13,14]. In particular, much attention has been paid to the potential use of colloidal gold nanoparticles (AuNPs) [15,16,17,18].

AuNPs are characterized by good solubility, a high ratio of active surface area to volume, the ability to modify the surface of the particles, and unique optical and electronic properties that depend on the size and shape of the nanoparticles. AuNPs can be synthesized in different size ranges and with different types of morphology [19]. The intense red color of the AuNPs dispersion is related to the interaction of the incident light, with a group of free electrons oscillating in the particles, a phenomenon known as localized SPR. Scientific reports confirm the possible application of functionalized spherical AuNPs to enhance the signal intensity in SPR-based research aimed at detecting the biomolecular interactions occurring at a flat metal‒solution interface [20]. Streptavidin (SA)-modified AuNPs and SPR imaging (SPRI) are used for the ultrasensitive detection of nucleic acids and are cost-effective compared to the nucleic acid amplification process, which is time-consuming as well as prone to sample contamination [21,22].

**Figure 1:**
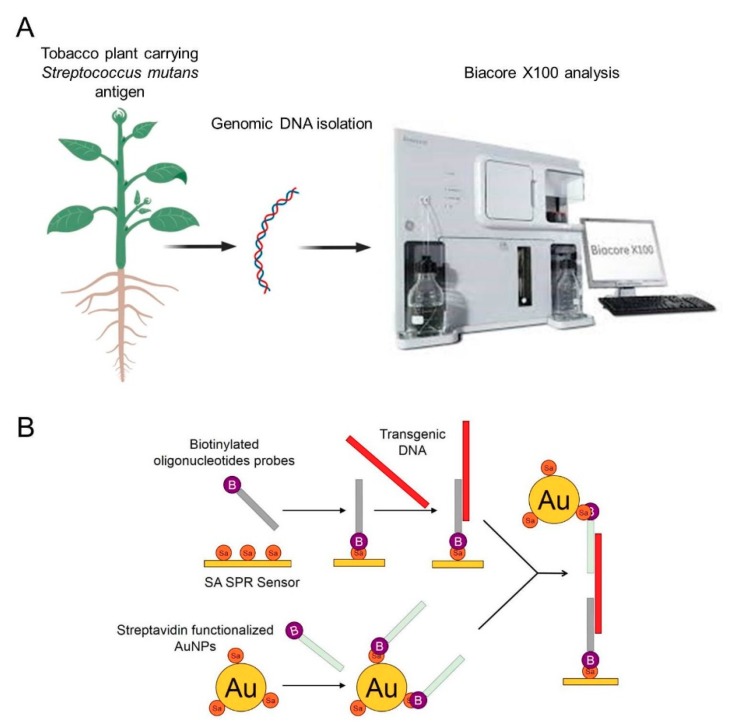
Schematic presentation of the experimental procedure. (**A**): general procedure of detection of transgenic plant using AuNPs based SPR biosensor, (**B**): schematic presentation of transgenic DNA detection using SA SPR sensor and AuNPs.

In the present study, a transgenic tobacco carrying a *Streptococcus mutans* antigen was used to design an SPR approach for the detection of foreign nucleic acid. Hybridization between the genomic DNA isolated from the leaves, stems, and roots of the transgenic tobacco and the biotinylated oligonucleotide probes immobilized onto an SA sensor chip was the basis for the detection of the target DNA. To increase the sensitivity, SA-functionalized AuNPs coated with a second type of biotinylated probe were applied. A schematic illustration of the experimental setup is shown in Figure 1. Transgenic DNA was detected rapidly and sensitively, which suggests that a unique SPR biosensing technique could be used to monitor the GMOs with high efficiency. An advantage of this technique is that it allows the use of nonamplified genomic DNA, which helps avoid the time-consuming step of amplification and possible sample contamination.

## 2. Materials and Methods

### 2.1. Synthetic Oligonucleotides

The nucleotide sequences of the biotinylated oligonucleotides (the AuNPs probe and SPR probe) and the PCR primers (Genomed, Warsaw, Poland) are provided in Table 1.

### 2.2. Tobacco Samples

Non-transgenic and transgenic tobacco expressing the surface antigen I/II of *S. mutans* were obtained from the Department of Biotechnology, Institute of Natural Fibers and Medicinal Plants in Poznań, Poland. Leaves, stems, and roots of tobacco were ground in liquid nitrogen, and genomic DNA was isolated using the DNeasy Plant Mini Kit (Qiagen, Hilden, Germany). The resulting DNA samples were analyzed quantitatively and qualitatively using a NanoDrop 2000c UV‒vis spectrophotometer (ThermoScientific, Waltham, USA). The DNA fragment was amplified using Taq DNA polymerase (Thermo Fisher Scientific, Waltham, USA) and SA I/II F and SA I/II R primers (Table 1). A total of 30 PCR cycles were performed. These cycles involved the following steps: denaturation—94 °C/45 s, annealing—60 °C/60 s, and synthesis—72 °C/60 s. The 300 bp PCR product separated on a 1.3% agarose (Sigma-Aldrich, Poznań, Poland) gel was used for the preparation of the positive control samples. The band corresponding to the mass of the PCR product was extracted from the gel and purified using a QIAquick Gel Extraction Kit (Qiagen, Hilden, Germany).

### 2.3. Synthesis of AuNPs

Citrate-stabilized AuNPs were synthesized by applying the citrate reduction method (Turkevich). Briefly, 2 mL of C_6_H_5_O_7_Na_3_ (38.8 mM) (Sigma-Aldrich, Poznań, Poland) was quickly added under vigorous stirring to 20 mL of a boiling aqueous solution of HAuCl_4_·3H_2_O (1 mM) (Sigma-Aldrich, Poznań, Poland). The color of the mixture changed from yellow to deep red, and a complete reduction was obtained after 10 min. The solution was then cooled to room temperature and filtered through a 0.45 μm membrane filter. The resulting colloidal solution was characterized by UV‒vis spectroscopy (NanoDrop 2000c), dynamic light scattering (DLS) (Zetasizer Nano ZS90, Malvern, UK), and transmission electron microscopy (TEM) (JEM-1400, JEOL, Tokyo, Japan).

### 2.4. Functionalization of AuNPs

AuNPs were functionalized according to the procedure described by D’Agata et al. [23]. Briefly, SA (Sigma-Aldrich) adsorption on the AuNPs surface was achieved by adding 10 µL of the SA solution (1 mg/mL in 10 mM phosphate-buffered saline (PBS) (Sigma-Aldrich, Poznań, Poland), pH 7.4) to 500 µL of colloidal gold solutions (5 nM, pH 8.5). After 1 h incubation on ice, the unreacted excess of SA was removed from the modified AuNPs-SA by centrifugation (30 min, 12500 rpm, 23 °C). Then, the supernatant was decanted, and the AuNPs-SA was dispersed in 90 μL of PBS. The resulting AuNPs-SA solution was incubated for 30 min with 10 μL of 100 µM biotinylated DNA probe (SA I/II AuNPs), followed by centrifugation (30 min, 12500 rpm, 23 °C) and removal of the supernatant. Finally, the AuNPs-SA-biotinylated DNA sample was dispersed in 50 µL of water. The obtained solution was characterized by UV‒vis spectroscopy, dynamic light scattering (DLS), and ζ-potential measurements.

### 2.5. SPR Experiments

The SPR experiments were performed on a Biacore X100 system (GE Healthcare Life Sciences, Pittsburgh, PA, USA) at 25 °C with HBS-EP+ as the running buffer. Instrument operations and data processing were carried out with the Biacore X100 Control software ver. 2.0 and Biacore X100 Evaluation software ver. 2.0, respectively.

Sixteen-nucleotide-long biotinylated DNA probes (SA I/II SPR Probe) were immobilized on an SA coated sensor chip (GE Healthcare). The SA chip was prepared by injecting a 120 µL conditioning solution (1 M NaCl, 50 mM NaOH) on the sensor. Next, 140 µL of 100 nM SA I/II SPR probe in HBS-EP+ buffer (GE Healthcare) was poured (flow rate 10 µL/min) for 1080 s over the sensor surface. The immobilization was performed using three different sensor chips, and the final level of immobilization was calculated and expressed in RU, where (1 RU = 1 pg/mm^2^).

The SPR signals were generated in the presence of the positive control sample and the denatured DNA samples that had sequences complementary to the SA I/II SPR probe, as well as the negative control sample. The samples with a concentration of 100 nM were injected at a flow rate of 10 µL/min over the sensor surface for 300 s. To remove the nonspecifically bound DNA, the sensor was washed with an HBS-EP+ buffer (flow rate 10 µL/min, 300 s). To amplify the signal of the SA I/II AuNPs probe, and functionalized AuNPs were used at the final stage of the SPR experiments. AuNPs at a concentration of 1 nM were injected (flow rate 60 µL/min) for 200 s, and the nonspecifically bound AuNPs were washed with the HBS-EP+ buffer (flow rate 10 µL/min, 300 s). The SPR response was recorded at 1100 s of the measurement, followed by the regeneration of the sensor surface. A single measurement cycle took 1500 s.

The regeneration conditions of the SA sensor were determined using a Regeneration Scouting Kit (GE Healthcare). The regeneration scouting process requires the injection of an analyte at high concentration (sample injection followed by AuNPs injection) and its dissociation from the ligand by applying a different regeneration solution, such as 10 mM glycine (pH 1.5–3), magnesium chloride (1–4 M), or sodium chloride (0.5–5 M), between the cycles. Each regeneration solution was tested by five repeated cycles of analyte injection followed by the injection of the regeneration solution.

### 2.6. Statistical Analysis

Data were acquired from at least three independent experiments and are reported as the mean ± standard deviation. Statistical differences were analyzed using the StatSoft Statistica 10 software (StatSoft Power Solutions, Inc., Tulsa, OK, USA) and factorial analysis of variance (ANOVA) with a post hoc Fisher’s least significant difference (LSD) test. Statistical significance was assumed for a p-value < 0.05.

## 3. Results and Discussion

### 3.1. Preparation of Plant Samples

**Figure 2:**
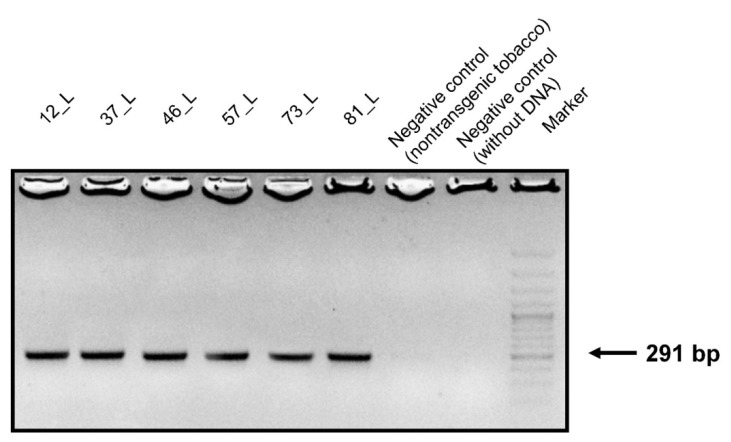
Agarose gel electrophoresis of the PCR products. Amplified DNA fragments of 291 bp from the leaves of transgenic tobacco fractionated on a 1.3% agarose gel. Size marker: GeneRuler™ 50 bp DNA ladder.

Genomic DNA samples were isolated from the leaves, stems, and roots of transgenic and non-transgenic tobacco. PCR primers were designed to contain a conserved sequence of the surface antigen I/II of *S. mutans*. DNA agarose gel electrophoresis confirmed that the conserved sequences of the 291-bp PCR product appeared only in the transgenic tobacco (Figure 2).

The amplified material was used for the preparation of the positive control samples. For this purpose, the bands corresponding to the mass of the PCR product were cut from the gel, purified, and used as reference samples in the SPR experiments.

### 3.2. Characterization of AuNPs

**Figure 3:**
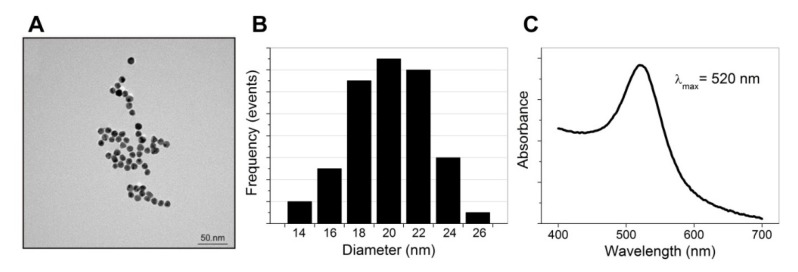
TEM image (**A**), size distribution (**B**), and UV‒vis absorbance spectrum (**C**) of the AuNPs.

The size, morphology, hydrodynamic diameter, and ζ-potential of the synthesized AuNPs functionalized with SA (AuNPs-SA) and the biotinylated DNA probe (AuNPs-SA-I/II) were investigated. Figure 3A shows TEM images of the bare AuNPs. The particles possessed a spherical morphology with an average particle size of approximately 20 nm (Figure 3B). The UV‒vis absorption spectrum exhibited a characteristic absorption peak for the bare AuNPs at 520 nm, as shown in Figure 3C. Table 2 presents the DLS measurements. The hydrodynamic diameter of the AuNPs was slightly larger than the diameter shown by the TEM images. The Z-average diameter of the AuNPs was 22.3 nm and increased after their functionalization with SA (40 nm). AuNPs-SA-I/II demonstrated a Z-average diameter of 44 nm. The further increase in the diameter corresponded to the contribution of the biotinylated DNA fragments to the hydrodynamic size after functionalization. Polydispersity index values indicated a narrow size distribution of the AuNPs. The high negative ζ-potential of the bare AuNPs and AuNPs-SA promoted the stability of the nanoparticles’ dispersion. The nanoparticles functionalized with the biotinylated DNA possessed a lower ζ-potential and were spontaneously scattered due to steric repulsion from the negatively charged oligonucleotides. It has been demonstrated that the adsorption of proteins on citrate-stabilized AuNPs involves carboxylate‒ammonium interactions established between citrate and the lysine or histidine amino groups on the protein’s surface, as well as steric or hydrophobic interactions with the nanoparticle surface’s adlayer [24]. The data presented by D’Agata et al. confirmed that the electrostatic repulsion forces due to the presence of the negatively charged oligonucleotide moieties promote the final stabilization of the AuNPs against aggregation [23].

### 3.3. I/II S. Mutans DNA-based Detection by SPR

#### 3.3.1. Immobilization of the Biotinylated SA I/II SPR Probe on the Sensor Chip Surface

**Figure 4:**
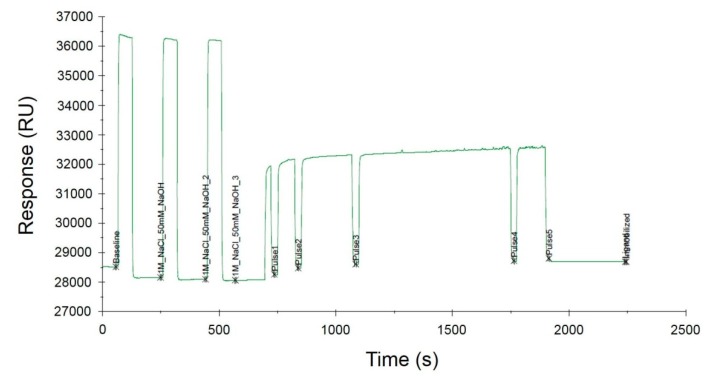
Immobilization of the biotinylated SA I/II SPR probe on the surface of the SA sensor chip.

SA I/II SPR probes were immobilized onto the surface of the SA sensor chip based on the strong interaction between biotin and SA. Immobilization of the biotinylated ligands on the SA-coated SPR sensor surface is a common approach that has been used for over a decade. This strategy is particularly recommended for the binding of nucleic acids [25,26] as it allows the orientation of the oligonucleotide probe sequence to be maintained, which is of great importance in the subsequent stages of SPR measurement. Immobilization occurred upon the injection of a 100 nM solution of biotinylated probes over the sensor surface covered with SA. DNA probes were immobilized on three different SA sensor chips, which resulted in the generation of three independent replicates. The immobilization levels were estimated to be 615.1 RU, 625.5 RU, and 630.8 RU, respectively, indicating a ligand density of ~620 pg/mm^2^, according to the conversion proposed by the manufacturer (1 RU = 1 pg/mm^2^). The representative sensorgram obtained during the immobilization process is illustrated in Figure 4. The sensor surface was prepared by three separate injections of the conditioning solution (1 M NaCl, 50 mM NaOH) for 730 s followed by controlled injection of the ligands. The Biacore X100 software precisely controlled the amount of the injected ligand while also monitoring the level of the SPR signal.

#### 3.3.2. Regeneration of the Sensor Chip Surface

**Figure 5:**
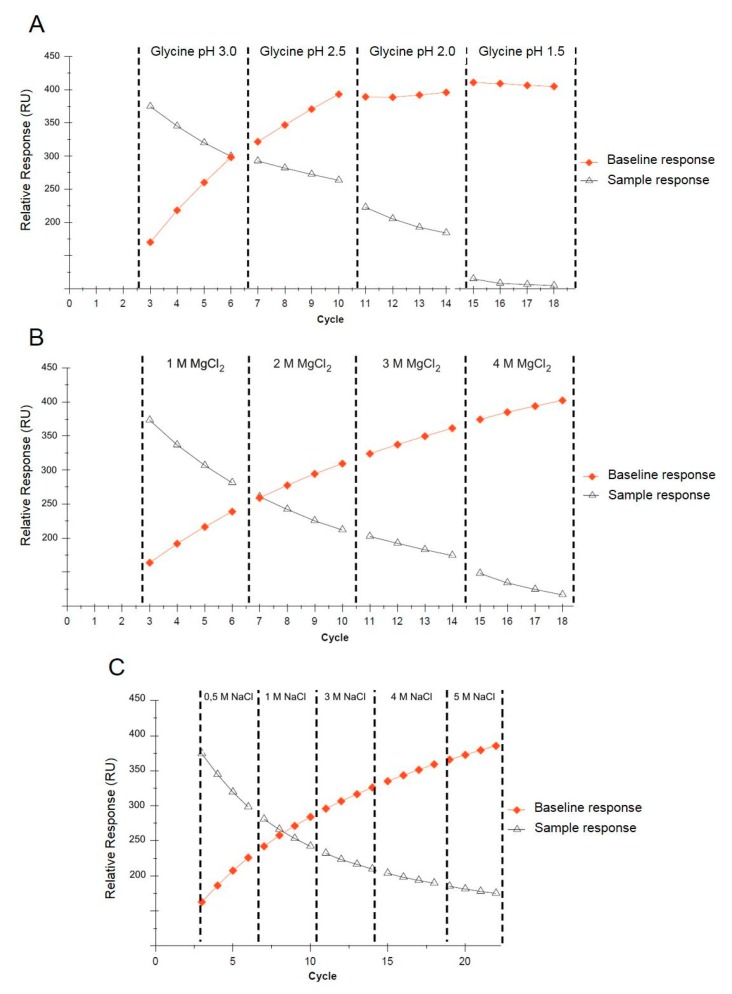
Trend plots of the baseline and analyte response levels against the cycle number, grouped according to the regeneration conditions using glycine (**A**), MgCl_2_ (**B**), and NaCl (**C**).

Multiple applications of a single sensor for reproducible SPR measurements require the selection of proper regeneration conditions. Regeneration should ensure the complete washing of the analyte from the sensor’s surface without any functional loss of the immobilized ligand. The regeneration conditions must be determined for each tested analyte, the immobilized ligand, and the sensor used in the experiment. After each SPR measuring cycle involving the injection of the positive control followed by interaction with the sensor surface and the application of the functionalized AuNPs, regeneration was performed. The sensor surface was washed with a glycine solution (according to pH) or magnesium chloride and sodium chloride (according to concentration). Trend plots of the baseline and sample response levels against the cycle number are shown in Figure 5. The best results were obtained for the glycine solution (pH 2.0), where an acceptable decrease in the response of the immobilized ligand throughout all four cycles was observed, whereas the baseline response remained constant (Figure 5A). Generally, a decreasing analyte response correlated with a stable baseline may indicate that the ligand is being denatured or dissociated from the surface, whereas a decreasing sample response correlated with an increasing baseline indicates that the analyte is not removed from the sensor surface. The results obtained for MgCl_2_ (1–4 M) and NaCl (0.5–5 M) were not satisfactory due to their very mild regeneration conditions (Figure 5B,C). A single SA sensor chip was used for approximately 90 measurements.

#### 3.3.3. Positive Control Sample Detection

**Figure 6:**
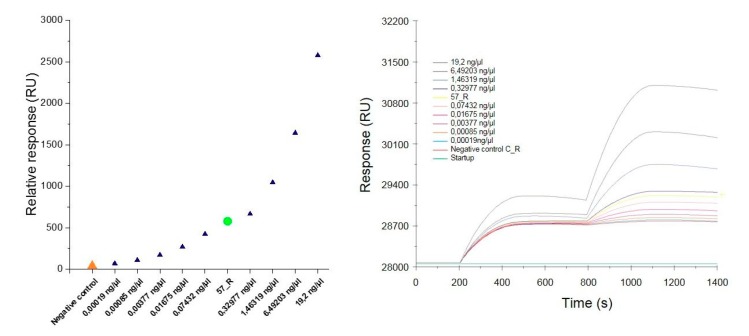
The relative SPR response against the concentration of the dilution of individual positive control samples and the DNA sample isolated from the roots of non-transgenic (C_R) and transgenic (57_R) tobacco (**A**). Overlay sensorgrams displaying the binding of the samples upon injection over the immobilized oligonucleotide probes followed by the enhancement of signal with the AuNPs-SA-I/II solution (**B**).

To determine the specificity and sensitivity of the constructed SA sensor, the positive control sample (PCR product extracted from the gel) was diluted to cover a concentration distribution from 100 nM to 1 pM. Then, the individual positive control samples were spread over the SPR sensor. After interaction of the samples with the immobilized complementary oligonucleotide probe, the sensor surface was washed with a buffer to remove the nonspecifically bound samples. Next, 1 nM of the functionalized AuNPs was applied to enhance the SPR response followed by washing to remove the unbound nanoparticles. The SPR response was recorded at 1100 s of the measurement. The results of the relative response plotted against the concentration of the dilutions of individual positive control samples and the DNA sample isolated from the roots of nontransgenic and transgenic tobacco (C_R and 57_R, respectively) are presented in Figure 6A and in Table 3. The response increased with the increasing concentration of the analyte. At the lowest concentration of 0.19 pg/µL corresponding to 1 pM, the response approached 70 RU. An interaction occurred upon injection of the complementary target (57_R), revealing a response of 578.5 RU. Only a slight increase was observed in response to the injection of the noncomplementary target (C_R) (40 RU). 

These results indicate that the constructed sensor chip was specific for the target analyte. Figure 6B displays the SPR response in terms of the adsorption of individual samples onto the immobilized oligonucleotide probes followed by the enhancement of the signal with AuNPs-SA-I/II. Due to the reduced sensitivity in identifying the hybridized DNA and RNA samples, it is necessary to amplify the signal using AuNPs [20]. As shown in Figure 6B, the response increased at 800 s, due to the injection of the functionalized AuNPs. Several approaches based on the use of AuNPs have been developed to amplify the response of the SPR [21,22,27,28]. DNA-modified AuNPs adsorbed onto the polyA tail on the miRNAs hybridized onto the locked nucleic acid (LNA) microarrays allowed detection at the attomole level when used in the SPRI method [27]. In the work by D’Agata et al., the ultrasensitive recognition of oligonucleotides (ODNs) was achieved by using a combination of surface-immobilized peptide nucleic acid (PNA) probes with continuous-flow microfluidics and nanoparticle-enhanced SPRI. The use of AuNPs combined with oligonucleotides complementary to the DNA target in the sandwich hybridization strategy enabled the differentiation of fully matched and single-base-mismatched sequences [21]. The same approach has been applied to enhance the detection of unamplified genomic DNA samples carrying different amounts of genetically modified sequences in Roundup Ready GMOs [22]. This biosensing system was able to selectively detect the genetically modified target sequence down to a zM concentrations in a mixture of genomic DNA even in the presence of a large excess of noncomplementary DNA. Wark et al. used a biosensing methodology called nanoparticle-enhanced diffraction gratings (NEDG), which combines the concepts of optical diffraction, planar SPR, and nanoparticle-enhanced detection methods to create a diffraction biosensor capable of identifying DNA at concentrations as low as 10 fM [28]. Biosensors with high selectivity can generate a more positive result after interaction with the target analyte compared to biosensors with poor selectivity, which tend to have a high false positive response. In this study, the selectivity of the biosensor is based on the highly conservative hybridization binding of the targeted sequence and the immobilized oligonucleotide probe (SPR probe). After injection of the negative sample, we observed no significant increase in response (40 RU). Additionally, the selectivity was increased by the application of AuNPs functionalized with oligonucleotide probes (AuNPs probe) complementary to the target sequence. The selectivity of the nucleic acid can, however, be hampered by nonspecific electrostatic interactions. A way to overcome this problem could be the application of PNA probes [22].

#### 3.3.4. Detection of Transgene Encoding an Antigen I/II of *S. Mutans*

**Figure 7:**
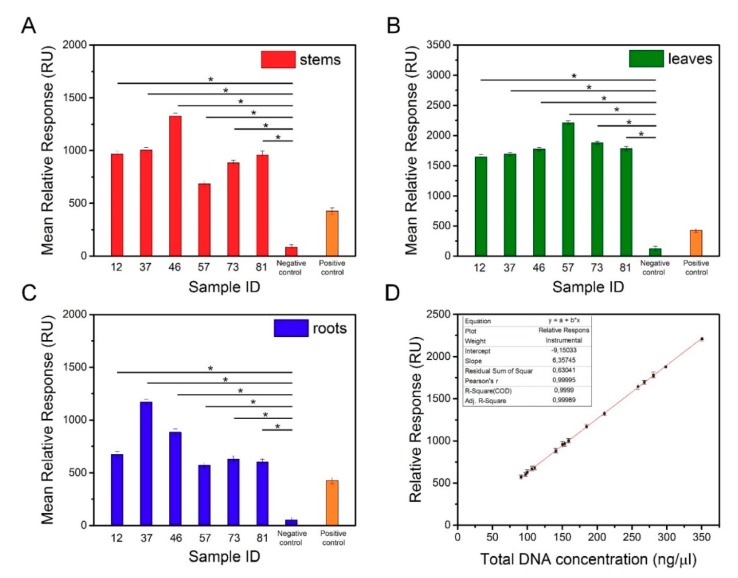
Mean relative responses of DNA samples isolated from the stems (**A**), leaves (**B**), and roots (**C**) of transgenic and non-transgenic tobacco. (**D**) Dependence of transgenic DNA concentration vs. relative responses.

Degenerated genomic DNA samples isolated from different organs of transgenic and non-transgenic tobacco were used in the SPR measurements. To avoid nonspecific interactions between the probes and genomic DNA, the sequences of biotinylated oligonucleotides were precisely designed to recognize the transgene (Table 1). Approximately 16 µL of the sample was needed to perform a single analysis using the Biacore X100 instrument. Experiments were conducted on three different SPR sensors with immobilization levels of 615.1 RU, 625.5 RU, and 630.8 RU. The concentration of the functionalized AuNPs used to amplify the SPR signal was 1 nM. To verify the obtained results, the positive control sample was used as a reference. The mean relative responses against the concentration of the analyzed DNA samples are presented in Table 4. The results of the SPR analysis in the replicated independent experiments (Figure 7A‒C) demonstrated that the average responses of the transgenic tobacco were significantly different from the responses of the nontransgenic tobacco. SPR-based biosensor technology is increasingly used for GMO detection. Biospecific interaction analysis in a Biacore system was applied to recognize the genetically modified gene sequences of Roundup Ready soybean, lectin, the 35S promoter, and the NOS terminator [29]. The results revealed a detection limit of 1 nM of the complementary fragments using the SPR system with tbiotinylated oligonucleotide probes immobilized on the sensor chip. In another study, an SPR method was developed to detect transgenic Cry1Ac cotton [30]. An SA-coated sensor chip was used to immobilize a 25-mer biotinylated probe, and the detection conditions were determined using Cry1Ac (230 bp) PCR products. The prepared sensor chip was capable of identifying the transgene at a concentration as low as 0.1 nM. However, in both studies, the target DNA fragments were amplified before the experiments, which extended the analysis time. In our work, we achieved the detection of nonamplified genomic DNA with a sensitivity as low as 1 pM. D’Agata et al. demonstrated the ultrasensitive recognition of non-amplified genomic DNA. In this study, a target sequence was identified by nanoparticle-enhanced SPRI using PNA probes [22]. Using this method, selective detection of the genetically modified sequence at a zM concentration in solutions comprising a mixture of nongenetically and genetically modified genomic DNA at an aM concentration (10‒30 pg/µL) is possible. The high efficiency of this biosensor results from the improved selectivity and sensitivity of the PNAs in recognizing the complementary DNA sequences, as well as the application of the functionalized AuNPs. As shown in Figure 7D, a regression equation enables estimation of the detection threshold for the transgene encoding the surface antigen I/II of *S. mutans*. At a concentration of 33 ng/μL, the SPR signal obtained for the sample containing the transgene was higher than the SPR signal of the negative control sample (nontransgenic).

## 4. Conclusions

In this study, a label-free and real-time SPR biosensing method using a Biacore X100 instrument was developed to detect foreign nucleic acid in genomic DNA samples without amplification. For this purpose, transgenic tobacco plants carrying an *S. mutans* antigen were used. Hybridization between the genomic DNA samples isolated from the leaves, stems, and roots of the transgenic tobacco and the biotinylated oligonucleotide probes immobilized onto an SA sensor chip were the basis for the detection of the target DNA. SA-functionalized AuNPs coated with a second type of biotinylated probe were used to increase the sensitivity of the measurements. Skipping the DNA amplification step significantly reduced the cost and time of the analysis. Thus, the results could be obtained much faster in comparison with conventional diagnostic methods, with limited demand for reagents. If this method is fully optimized, then the presence of a transgene in a genomic DNA sample could be verified within a few minutes.

## Figures and Tables

**Table 1:** Polymerase chain reaction (PCR) primers and surface plasmon resonance (SPR) probes.

	Sequence (5’→3’)
SA I/II F primer	TCTTGGTAGATCCCCTGCCA
SA I/II R primer	CAGGAGTTGTCACCCGAACA
SA I/II AuNPs probe	ATAAATCGTTG-Biotin
SA I/II SPR probe	Biotin-GTACAAGAACTTGTCC

**Table 2:** Data from TEM (physical diameter), DLS (hydrodynamic diameter), and ζ-potential characterization of AuNPs.

Sample	Physical Diameter (nm)	Hydrodynamic Diameter (nm)	ζ-potential (mV)	PDI
AuNP	20 ± 5	22.3	−38.0 ± 4	0.090 ± 0.012
AuNP-SA	-	40.0	−41.8 ± 4	0.235 ± 0.023
AuNP-SA-I/II	-	44.0	−9.4 ± 4	0.331 ± 0.003

**Table 3:** Results of SPR measurements for the dilutions of individual positive control samples and DNA sample isolated from the roots of nontransgenic (C_R) and transgenic (57_R) tobacco.

Sample ID	Concentration (ng/µL)	RelResp
1	0.00019	70.0
2	0.00085	109.7
3	0.00377	172.2
4	0.01675	270.3
5	0.07432	424.4
6	0.32977	666.2
7	1.46319	1045.9
8	6.49203	1641.9
9	19.2	2578.0
Startup	-	0.0
C_R	145.38	40.0
57_R	90.89	578.5

**Table 4:** Results of the SPR measurements for the DNA sample isolated from the stems, leaves, and roots of non-transgenic (C) and transgenic tobacco on the SA sensor, with an immobilization level of 615.1 RU.

Sample ID	Concentration (ng/µL)	Mean RelResp ± SD (RU)
12_S	153.53	965.4 ± 28.9 1644.5 ± 35.1 672.3 ± 29.0
12_L	258.46	
12_R	106.57	
37_S	158.95	1003.4 ± 24.9 1693.9 ± 24.9 1170.5 ± 25.1
37_L	267.72	
37_R	184.87	
46_S	210.42	1326.1 ± 26.8 1774.1 ± 26.7 884.2 ± 28.7
46_L	280.67	
46_R	140.5	
57_S	110.4	684.5 ± 25.5 2213.7 ± 29.5 570.8 ± 25.4
57_L	350.21	
57_R	90.89	
73_S	140.85	883.5 ± 25.1 1879.8 ± 24.0 628.1 ± 29.4
73_L	298.14	
73_R	99.84	
81_S	150.42	958.4 ± 32.4 1781.9 ± 33.0 602.3 ± 25.0
81_L	280.43	
81_R	97.21	
C_S	194.32	83.4 ± 23.3 125.0 ± 33.0 51.5 ± 24.7
C_L	280.83	
C_R	145.38	
Positive control	0.074	427.1 ± 31.0
